# Functional Roles and Genomic Impact of Miniature Inverted-Repeat Transposable Elements (MITEs) in Prokaryotes

**DOI:** 10.3390/genes15030328

**Published:** 2024-03-03

**Authors:** Michael F. Minnick

**Affiliations:** Program in Cellular, Molecular and Microbial Biology, Division of Biological Sciences, University of Montana, Missoula, MT 59812, USA; mike.minnick@mso.umt.edu

**Keywords:** prokaryote, genomes, miniature inverted repeat transposable elements, MITEs, evolution, transposons, Tns, non-autonomous, mobile genetic elements, MGEs

## Abstract

Prokaryotic genomes are dynamic tapestries that are strongly influenced by mobile genetic elements (MGEs), including transposons (Tn’s), plasmids, and bacteriophages. Of these, miniature inverted-repeat transposable elements (MITEs) are undoubtedly the least studied MGEs in bacteria and archaea. This review explores the diversity and distribution of MITEs in prokaryotes and describes what is known about their functional roles in the host and involvement in genomic plasticity and evolution.

## 1. Introduction

The discovery of “jumping genes” (transposable genetic elements or transposons) in maize by Barbara McClintock in the 1940s was monumental for its contribution to our understanding of the dynamic nature of the genome. From McClintock’s work, it was clearly apparent that phenotypic traits were not dictated solely by Mendelian genetics, and that gene linkage was not fixed within a chromosome. Rather, phenotypes could be changed stochastically, gene linkages rearranged, and gene expression altered as a result of insertion or loss of transposable elements (TEs) [1,2]. Although the tremendous significance of McClintock’s research was not recognized for over thirty years, it eventually earned her a Nobel Prize in Physiology or Medicine in 1983. Today, the role of TEs in genomic plasticity and evolution is universally accepted dogma. Other mobile genetic elements have since been discovered, and together with TEs constitute a “mobilome” that is continually in flux and manipulating the genomic makeup of organisms in all three domains of life. 

**Figure 1:**
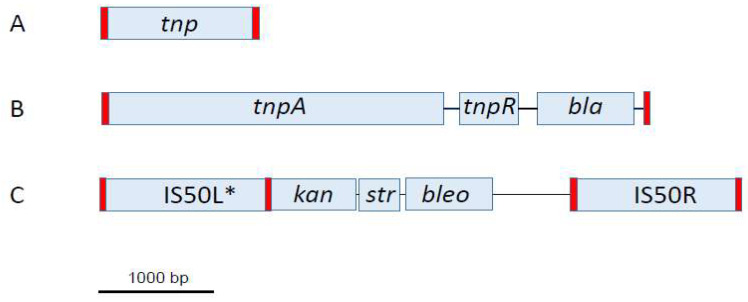
Examples of the three types of TEs. (**A**) Insertion sequence IS2 from *Escherichia coli*. The IS2 *tnp* gene consists of two ORFs (*orfA* and *orfB*) that encode the active transposase, which is produced following a -1 translational frameshift and fusion. (**B**) Simple transposon Tn3 from *E. coli* possessing *tnpA* (transposase), *tnpR* (resolvase), and *bla* (β-lactamase) genes. (**C**) Composite transposon Tn5 containing two flanking IS50 elements plus genes conferring resistance to kanamycin (*kan*), streptomycin (*str*), and bleomycin (*bleo*). * IS50L of Tn5 is defective for transposition due to a nonsense mutation in its transposase gene. Red rectangles indicate the positions of TIRs.

The basic structure of a TE includes a set of terminal inverted repeats (TIRs) that surround a transposase (*tnp*) gene whose encoded enzyme mobilizes the element. The simplest TE is the insertion sequence (IS), and it consists solely of this basic structure. As such, IS elements are relatively small (~800–2000 bp) with TIRs of ~10–50 bp (Figure 1A). If the TE includes the basic structure plus accessory genes (e.g., antibiotic resistance markers, virulence genes, etc.), it is referred to as a simple TE. Such elements can reach several thousand base pairs in length, depending on their gene content (Figure 1B). Finally, when two IS elements flank a set of accessory genes, a composite TE is formed (Figure 1C). Here, the transposase is encoded by one or both of the flanking IS elements, rather than within the intervening core sequence, as observed in IS elements and simple TEs. 

Transposition is an autonomous process catalyzed by enzymes encoded within the respective element, and TEs can be transposed by one of two methods. TEs that use a RNA intermediate as a template and reverse transcriptase to generate double-stranded DNA for re-integration into the genome undergo a “copy and paste” method of mobilization and are referred to as Class I TEs or retrotransposons. Nearly all known retrotransposons occur in eukaryotes. In contrast, TEs that employ a “cut and paste” method with a DNA intermediate are referred to as Class II TEs or DNA transposons. DNA transposons occur in both prokaryotes and eukaryotes and are the type relevant to this review. 

Mobilization of DNA transposons involves transposase-mediated: (1) excision of the element at its TIRs, (2) cutting of the target DNA sequence to yield sticky-end overhangs, and (3) ligation of the TE into the target site. Following integration, host DNA polymerases are recruited to repair any gaps that surround the new insert. Target site specificity of the mobilization process varies with the kind of TE. For instance, Tn7 targets a single specific site in the bacterial chromosome at *att*Tn7 [3], whereas many other TEs randomly target host DNA. 

Two general types of transposition events are known. If a Tn is completely excised and mobilized to a different location in the genome, it is referred to as conservative transposition, since the process ensures maintenance of a single copy of the element in the cell. This kind of transposition is observed in vitro with Tn5 (Figure 1C) [4]. Alternatively, if the TE is replicated and the resulting copy mobilized, it is referred to as replicative transposition. The net effect of this mechanism is to maintain a copy of the element in its original locus plus at least one other integration site in the chromosome. Replicative transposition is observed with Tn3 (Figure 1B) [5].

While the ability to mobilize in a genome is a hallmark characteristic of TEs, not all elements can do so autonomously. For example, if mutations generate a defective transposase gene, the resulting element can only be mobilized in trans through transposase enzymes supplied by similar, or distantly related, intact transposons. Such mutated versions of TEs are referred to as miniature inverted-repeat transposable elements (MITEs) in reference to their relatively smaller size (50–500 bp) due to sequence decay of the non-functional transposase and/or other core sequences. MITEs have been investigated to a much greater degree in eukaryotes than prokaryotes, especially in plants. Adaptive roles ascribed to MITEs of eukaryotes include serving as a source of microRNAs for gene regulation and adaptation [6,7], modulation of gene expression in nearby genes [8], influencing alternative RNA splicing events (exonization) [9], and driving genome complexity and allelic diversity through transposition [10].

## 2. Prokaryotic MITEs 

### 2.1. Conserved Structural Features

**Figure 2:**
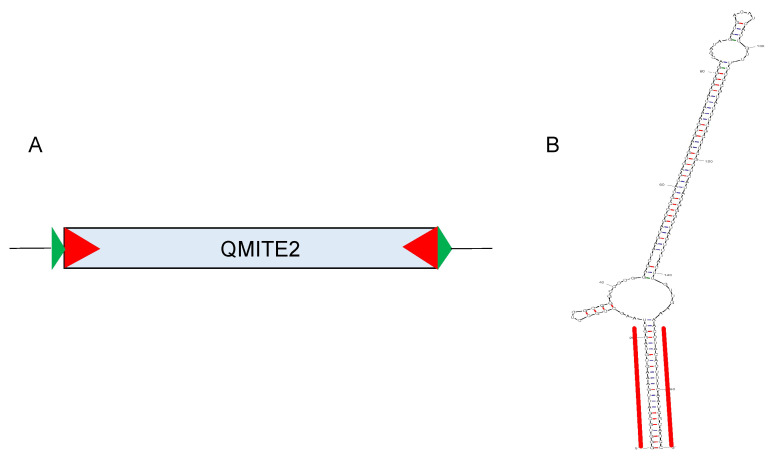
A representative prokaryotic MITE. QMITE2 of *C. burnetii* is depicted (adapted from [14]). (**A**) A model of QMITE2 including its DRs (7–9 bp, green), TIRs (23–29 bp, red), and a core sequence (113–145 bp, blue) is shown. (**B**) Secondary structure prediction of the full-length QMITE2 transcript by mFold [15] showing TIRs (red lines) and a ∆G value = −113.09 cal/mol.

Prokaryotic MITEs share the same basic architecture as eukaryotic MITEs, with a total length typically <500 bp, TIRs of ~4–30 bp, and a flanking set of direct repeats (DRs) in relatively “younger” MITEs, following duplication of the target sequence [11,12]. Of note is that maintenance and conservation of the DRs wanes over time [11]. MITEs are typically AT-rich, tend to integrate into AT-rich intergenic regions of the genome, and produce transcripts that form thermodynamically stable, stem-loop secondary structures [13]. A representative MITE from *Coxiella burnetii* (QMITE2) and the predicted secondary structure of its transcript are shown in Figure 2 [14]. 

IS elements are thought to be the main source of MITEs in prokaryotes. This is not surprising since ISs are the most common type of autonomous TE in these organisms, and they can catalyze the mobilization of related MITEs in trans [16,17,18]. 

### 2.2. Size and Sequence Variation

There are two general categories of MITEs. Type I MITEs are those that share considerable sequence conservation with related, autonomous IS elements, while type II MITEs share sequence conservation that is strictly confined to their TIR regions [19]. In actuality, these designations are somewhat arbitrary, as a range of degeneracy from autonomous, recently-transposed IS elements to related, but “older”, type II TEs could co-exist at any point in time in an organism. Such a gradient is clearly observed in the *C. burnetii* QMITE1 and QMITE2 families [14]. For instance, in the Nine-Mile type strain of *C. burnetii* (RSA493), it was found that the chromosome possessed 45 QMITE1 elements ranging in size from 39 to 400 bp. Roughly half of these MITEs were 39 to 321 bp long and shared <75% sequence identity with the full-length (~400 bp) QMITE1, although their TIRs and DRs showed a relatively higher degree of conservation. A second family, QMITE2 (Figure 2), demonstrated even greater size and sequence variation. Of the 78 copies of QMITE2, only ten were “full-length” (~190 bp) MITEs. Of the remaining elements, 12 copies showed a loss of ~40 bases at their 5′ ends. Notably, the loss of DRs and TIRs in these markedly truncated copies produced elements that more closely resembled repetitive extragenic palindromes (REPs) than typical MITEs [14]. 

The *Chunjie* MITE of *Geobacter uraniireducens* also demonstrates considerable diversity with respect to size and sequence. For example, full-length *Chunjie*-1 is a 235-bp element that disrupts one copy of the bacterium’s two phosphate uptake operons. Thirty-seven additional copies of the element, with conserved TIRs and DRs but ranging in size from 178 to 235 bp, are located elsewhere in intergenic regions of the genome. Remarkably, BLAST searches revealed yet another 57 copies of partial *Chunjie* elements, including presumably inactive elements lacking TIRs or DRs [11].

## 3. Distribution across Prokaryotic Genomes

### 3.1. Taxonomic Distribution

The first prokaryotic MITE was discovered in *Neisseria gonorrhoeae* and *Neisseria meningitidis* in 1988. Named after a discoverer, the 204-bp Correia element was found in both species with an estimated 20 copies each [20]. This number was later revised upwards to >120 copies of the MITE based on the *N. gonorrhoeae* genome sequence [21]. Following the discovery of Correia in *Neisseria*, MITEs have been found in numerous other bacteria, including members of the *Enterobacteriaceae* family [22,23], cyanobacteria [12,24,25], *Staphylococcus aureus* [26], *Acinetobacter* [27,28], pseudomonads [29,30], *Porphyromonas* [31,32], *Polaromonas* [33], and an endosymbiont of spiders [34]. In fact, MITEs appear to be a universal feature of bacteria. Although relatively less is known about the MITEs of archaea, they have been identified in *Sulfolobus* spp. [35,36,37], *Halobacterium* [38], *Haloquadratum walsbyi* [12], *Delsulfurococcus kamchatkensis* [39], and several other archaeal genomes [40], to date. Interestingly, the archaeon *Methanobacterium thermoautotrophicum* is unusual for its lack of any apparent MITEs [38]. 

### 3.2. Copy-Number Variation

While all MITEs tend to be multicopy in nature, the actual number in a genome varies considerably per given host and MITE family. This is perhaps best exemplified in the MITEs of plants, where an astounding 8–10% of the genome can be comprised of elements representing dozens of MITE families infecting a single plant species, and arising from single or numerous rounds of multiplication [9,41]. While nothing to this extreme has been observed in prokaryotes, MITE copy numbers can still be high and variable. For example, the MITE*_Aba12_* and its orthologs vary from 1 to 22 elements per genome, depending on the host *Acinetobacter* species and strain examined [28]. In the archaeon *Sulfolobus solfataricus* P2, four MITE families (designated SM1–SM4) exist and range in copy numbers from 25 (SM2) to 44 (SM3) [42]. Finally, the copy number of the enterobacterial repetitive intergenic consensus (ERIC) MITE found in the *Enterobacteriaceae* and *Vibrionaceae* families varies considerably, with copy numbers correlating with sub-family taxa [43]. ERIC genomic estimates of 30 copies in *E. coli* (strain K-12), 150 in *Salmonella enterica* (Typhimurium LT2), and 711 copies in *Photorhabdus luminescens* have been reported [22,44,45]. As an example of strain-level variation, 14 additional ERIC elements have been identified in novel intergenic regions of genomes from *E. coli* strains other than K-12 [44].

### 3.3. Insertion Site Preferences

MITEs of prokaryotes, like their eukaryotic counterparts, are normally located in intergenic regions of the genome. For example, the BrickBuilt MITE of *Porphyromonas gingivalis* and the *Caulobacter* CcrM-associated intergenic repeat (CIR) element of *Caulobacter crescentus* were reported to be strictly intergenic [32,46]. Similarly, MITEs derived from the ISSoc2 of *Synechococcus* were located intergenically or within genes of unknown function [47], and QMITE 2 of *C. burnetii* was primarily found in the 3′ untranslated region of ORFs [14]. While these locations are typical, exceptions exist. For example, even in ERIC elements, where the “I” of the acronym actually denotes “intergenic”, examples of intragenic locations have been reported [48]. Presumably, any integration event that could confer a selective advantage on the host through the functional role(s) of a particular MITE would be favored and the element retained. Moreover, MITE insertion into intergenic regions would have no chance for deleterious gene inactivation; however, it could still play a role in genomic plasticity (see Section 4.2, below). Interestingly, *Streptococcus pneumoniae*’s BOX element, repeat units of pneumococcus (RUP), and *S. pneumoniae* rho-independent terminator-like element (SPRITE) were frequently found bunched together in the chromosome, with elements even inserting into one another. These “clusters” were found together with MITEs in more typical intergenic regions, including regulatory sequences and pseudogenes [49]. 

## 4. Functional Roles and Genomic Impact

### 4.1. Influence on Gene Expression 

Early work with Correia showed that a σ^70^-like promoter sequence on the element could serve as a transcriptional promoter for the *N. gonorrhoeae uvrB* gene when expressed ectopically in *E. coli uvrB* mutants [50]. In fact, subsequent work showed that subtypes of the Correia element encoded two strong promoters that could enhance the expression of proximal genes [51]. Clearly, the predominant mapped locations of Correia immediately upstream of genes would provide an optimal arrangement for promoter function [21]. Work has also shown that the Correia element can undergo inversion, especially under conditions where the host is under antibiotic stress [52]. Considering the endogenous promoters that occur in Correia, this kind of mobilization would certainly affect the expression of nearby genes and might play a role in the reversible phase variation seen in *N. gonorrhoeae* [52]. Since the earlier work with Correia, σ^70^-like promoter sequences have also been discovered in many other kinds of prokaryotic MITEs, including *Acinetobacter* MITEAba12 [28]; *Porphyromonas* BrickBuilt [32], *Coxiella* QMITE1 [14], and *Shewenella* SonMITE_1 [53].

Another means by which MITEs can regulate expression is by encoding an integration host factor (IHF)-binding site. IHF is a nucleoid-associated protein that can bind cognate IHF sites of 30–35 bp in length and subsequently bend the underlying DNA in order to optimize a variety of biological functions, including transcription [54]. A bona fide IHF-binding site was previously discovered in a Correia element inserted immediately downstream of the promoter for the *mtrCDE* efflux pump operon of *N. gonorrhoeae*. When the IHF-binding site was deleted, transcription of the operon was upregulated in vivo, clearly demonstrating a regulatory role for the MITE embedded in the sequence [55]. 

A third way that MITEs can post-transcriptionally regulate expression is through RNase-mediated cleavage of a transcript containing the particular element. This was demonstrated with *mtrCDE* transcripts of *N. gonorrhoeae* containing a Correia element, where RNase III cleaved the mRNA at the TIRs [55]. Similar RNase III processing has also been reported for other kinds of transcripts containing Correia elements [56,57]. Interestingly, the insertion of ERIC elements at the 3′ end of *Yersinia enterocolitica* genes can also affect levels of the respective ERIC-containing transcripts, by unmasking a RNase E site in the 3’ TIR and enhancing the mRNA’s degradation [58]. 

A fourth way that MITEs can regulate expression is through insertional inactivation of genes. Recent work with *Acinetobacter baumannii*, a highly adaptable, multidrug-resistant bacterial pathogen, demonstrated the active mobilization of MITEaba12 into a histone-like nucleoid structuring (*hns*) global regulator gene in response to desiccation stress [28]. Interestingly, the *hns* locus is apparently a “hotspot” for insertional inactivation by various TEs in *A. baumannii* during stress, and loss of H-NS function following insertion generates high-level colistin resistance, hypermobility, and other phenotypic changes [59,60]. 

A fifth way that MITEs could conceivably regulate expression is through their production of non-coding small regulatory RNAs (sRNAs). Much like microRNAs originating from the MITEs of plants (reviewed in [61]), sRNAs encoded by prokaryotic MITEs could possibly play a role in prolonging or decreasing a target mRNA’s longevity or by regulating translation following sRNA:mRNA complex formation. Of course, these activities would depend on the physical location of the sRNA:mRNA interaction(s). Production of sRNAs by MITEs has been reported in *C. burnetii*, where two sRNAs, Cbsr3 and Cbsr13, were found to originate from QMITE1, while Cbsr16 originated from QMITE2 [14]. The biological role and mRNA targets for these three sRNAs is unknown but intriguing. 

Finally, it is conceivable that MITES could also regulate expression through the formation of riboswitches or Rho-independent transcriptional terminators in the mRNAs where they occur. As mentioned above, the characteristic formation of stable secondary structures in MITE transcripts would clearly be amenable to the formation of such regulators. The occurrence of Rho-independent terminator-like sequences in SPRITE elements and potential T-box riboswitch motifs in BOX elements of *S. pneumoniae* underscores these possibilities [18]. 

### 4.2. Role in Genome Plasticity and Evolution 

Genome plasticity is fundamental to successful evolution, and one of the most basic mechanisms is the acquisition of novel genes encoded by the MGEs themselves. Moreover, as these elements are mobilized and integrated, there is an increased likelihood of evolution through gene duplication and/or merodiploidy in the host. Research on prokaryotic MITEs has shown that they often encode novel ORFs, presumably derived from progenitor IS elements or acquired through recombinational events. For example, several MITEs of enteric bacteria, including ERIC, were found to possess ORFs [48]. In many instances, the ORFs within MITEs have been found to encode proteins or peptides of unknown functions or that lack obvious homologs in the databases. Examples include the large BOX elements of pneumococcus [49], intragenic ERIC elements [48], QMITE1 of *C. burnetii* [14], and MITEb and MITEe of *Anabaena* sp. strain PCC 7120 [62]. 

While it is not unusual for ORFs in intragenic MITEs to generate in-frame fusions with genomic ORFs upon their integration, *Rickettsia* palindromic elements (RPEs) appear to have taken the situation to an unusually high level of frequency. As first reported, RPEs of ~150 bp, representing ten identified RPE families, integrated 44 times in unrelated metabolic genes of *Rickettsia conorii*, the agent of Mediterranean spotted fever [63]. Each RPE was found to encode an ORF with a conserved peptide. Nearly half the RPEs, from three of the ten families identified, were inserted into genomic ORFs with the ability to modify the underlying gene product through a translational fusion. A dozen homologs of the RPEs were also found in genomes of *Rickettsia felis*, *Rickettsia prowazekii*, and *Rickettsia helvetica*, and eleven of the twelve elements were similarly inserted into genomic ORFs in an in-frame manner [63,64]. To what extent these insertions have contributed to the evolution of novel or modified proteins of *Rickettsia* spp. remains unexplored, but the predicted compatibility between three-dimensional protein conformation and function following RPE modification is intriguing [64]. Moreover, it is conceivable that a similar MITE-mediated mechanism of protein evolution has occurred in other prokaryotes.

MITES also play an important role in genome reduction through insertional inactivation of genes that are no longer needed by the host prokaryote. Together with autonomous TEs, this mechanism of purifying selection significantly contributes to genomic plasticity. Typically, there are many more MITEs per genome relative to the corresponding, autonomous precursor elements, suggesting that MITEs may play a significantly greater role in genome plasticity [65]. Insertional inactivation of genes by MITEs has been demonstrated in a variety of prokaryotes. For example, the cyanobacterial microcystin toxin gene cluster, *mcy*, was shown to be functionally inactivated by MITEs in two naturally-occurring strains of *Anabaena* collected from the Baltic Sea [66]. Interestingly, the generation of non-toxic, microcystin-negative strains of cyanobacteria commonly occurs in both laboratory and natural settings, and it is tempting to suggest that MITEs may be responsible for this widespread phenotypic variation [66]. In another example, comparative genomics work on *N. gonorrhoeae* has demonstrated that the Correia element differentially disrupts coding sequences in a strain-dependent manner, underscoring the MITE’s mobility and utility in “shaping” the pathogen’s genome [21]. In *Ralstonia solacearum* and *Pseudomonas syringae*, two notorious plant pathogens, MITEs have been shown to insert and inactivate the type III secretion system effector genes *avrA* and *hopX1* (*avrPphE*), respectively [29,67]. These insertional inactivation events are thought to modulate a particular strain’s virulence and expand its host range for certain plants. Finally, in the archaeon *S. solfataricus*, a type II MITE has been shown to insertionally inactivate the *pyrE* gene (encoding orotate phosphoribosyl transferase) in certain naturally-occurring strains, demonstrating the element’s ability to mobilize in vivo. The biological significance of this *pyrE* inactivation in natural populations is unknown but certainly intriguing [68]. 

**Figure 3:**
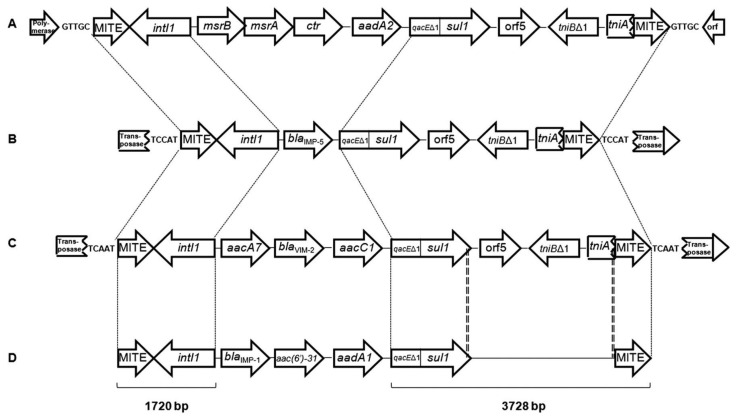
Depiction of various class I integrons of *Acinetobacter* spp. flanked by identical, MITEs (reprinted from [73] by permission). (**A**) strain NFM2 of *A. johnsonii* isolated from prawns; (**B**) strain 65FFC of *A. baumannii*; (**C**) strain 118FFC of the nosocomial pathogen, *A. bereziniae*; and (**D**) strains 694-696, 5227, 5248 and 9043 of *A. baumannii*. Nucleotide sequences were identical between dashed lines in (**A**–**C**), whereas the region between double-dashed lines in (**D**) was verified by size conformity between PCR products compared to (**A**–**C**) [73]. Arrows denote gene cassettes encoding identical MITEs (MITE); methionine sulfide reductase (*msrA*/*B*), integrase (*intI1*), transposase (*tniA*/*B*∆*1*), and resistance genes (all others).

MITEs have also been shown to mobilize antimicrobial resistance genes in bacteria, and this activity would clearly be adaptive to pathogens or soil microbes that are routinely exposed to antibiotics. For example, early work with E622, a 611-bp MITE involved in *P. syringae* virulence, demonstrated that it was able to mobilize a β-lactamase (*bla*) gene [30]. More recently, the *mcr-5* colistin resistance cassette was shown to be mobilized in trans by the IR148 MITE of *E. coli* using a transposase provided in trans by *TnAs1* [23]. Finally, intriguing work has implicated MITEs in the mobilization of integrons; a common bacterial genetic element that provides for the acquisition and utilization of multiple gene cassettes encoding various gene products, including virulence factors, metabolic enzymes, and antimicrobial- or disinfectant-resistance determinants [69]. For example, a class I integron encoding a *bla* gene for imipenem resistance in a clinical isolate of *A. baumannii* was found to possess identical MITEs at both of its termini [70]. The exact same MITEs have also been found flanking a Class I integron of *Acinetobacter johnsonii* isolated from the gut of a prawn, with cassettes encoding streptomycin-resistance and methionine sulfoxide reductase [71]. Finally, additional work has discovered identical MITEs flanking different class I integrons in seven *Acinetobacter* spp., and one unique integron from *Acinetobacter bereziniae* possessed *aacC1* and bla_VIM-2_ genes for aminoglycoside and carbapenem resistance, respectively (Figure 3) [72,73]. 

Taken as a whole, the existence of identical MITEs surrounding disparate integrons in various strains and species of *Acinetobacter* suggest that these elements have played a significant role in class I integron mobilization, thereby facilitating the acquisition of antimicrobial/disinfectant resistance and novel physiological traits within members of the genus. It is quite likely that MITE-mediated mobilization of integrons has also occurred in other bacteria where both MITEs and integrons are present.

Like IS elements, MITEs serve as important hotspots for homologous recombination with the chromosome during evolution. Likewise, stretches of genomic sequences flanked by homologous MITEs can be mobilized to new locations. A clear example is seen in *C. burnetii*, where QMITE2 once served as a target for transposition by IS1111, through the IS element’s recognition of the MITE’s GTAG repetitive extragenic palindrome [14]. Specifically, the two IS1111 TEs have integrated into the two QMITE2 copies that flank the chromosomal *icm*/*dot* genes for the type IV secretion apparatus [14]. Mobilization and acquisition of the *icm*/*dot* virulence determinant would clearly have been advantageous to *C. burnetii* over the course of its evolution to become an obligate intracellular parasite, and MITEs apparently played a role [74]. 

## 5. Bioinformatic Tools for MITE Analysis

As of the time of writing, six open-source software programs were freely available online to identify putative MITEs in sequence datasets. From the most recent to the earliest, these include MITEFinder II [75], MITE Tracker [76], MUSTv2 [77], detectMITE [78], MITE Digger [79], and MITE-Hunter [80]. Not surprisingly, these algorithms initially search for potential MITEs in genomic sequence data using their conserved structural features, including TIRs and DRs (i.e., target-site duplications; TSDs). Low-complexity TIRs are typically filtered out to decrease false positives based on a set of selection criteria such as low sequence identity and similarity to MITEs in the Repbase database. Sequence alignments are also employed to determine sequence identity, allowing for mismatches, to determine the relatedness of the potential elements to known MITEs, and to group potential MITEs into families. Major issues that have confounded software developers include the upper size limit for the input sequence and the high rate of false positives identified, although this problem has apparently been minimized with MITEFinder II, relative to other algorithms [75].

## 6. Conclusions and Future Directions

Prokaryotic MITEs are non-autonomous mobile genetic elements that shape and “flex” the genomes of host archaea and bacteria through their ability to be mobilized in trans by autonomous TEs. The resulting transposition event can introduce novel ORFs, insertionally inactivate genes no longer needed, and relocate sections of the genome, thereby facilitating evolution. Following integration, MITEs remain active participants in the host’s biology through adaptive roles in transcriptional regulation, protein evolution, and post-transcriptional regulation by their encoded sRNAs. While several reports on the functional roles of MITEs in eukaryotic systems have been published, to date, relatively few studies exist for prokaryotic MITEs. As such, further investigation of prokaryotic MITEs should provide fertile ground for discovery.

## Abbreviations

CIR (*Caulobacter* CcrM-associated intergenic repeat); DRs (direct repeats); ERIC (enterobacterial repetitive intergenic consensus); IHF (integration host factor); IS (insertion sequences); MGEs (mobile genetic elements); ORF (open reading frame); REPs (repetitive extragenic palindromes); RPEs (*Rickettsia* palindromic elements); RUP (repeat units of pneumococcus); SPRITE (Streptococcus pneumoniae rho-independent terminator-like element); TEs (transposable elements); TIRs (terminal inverted repeats); Tn (transposon); TSDs (target site duplications); MITEs (miniature inverted-repeat transposable elements).
